# *Hwanggeumchal sorghum* Induces Cell Cycle Arrest, and Suppresses Tumor Growth and Metastasis through Jak2/STAT Pathways in Breast Cancer Xenografts

**DOI:** 10.1371/journal.pone.0040531

**Published:** 2012-07-06

**Authors:** Jin Hee Park, Pramod Darvin, Eun Joung Lim, Youn Hee Joung, Dae Young Hong, Eui U. Park, Seung Hwa Park, Soo Keun Choi, Eon-Soo Moon, Byung Wook Cho, Kyung Do Park, Hak Kyo Lee, Myong-Jo Kim, Dong-Sik Park, Ill-Min Chung, Young Mok Yang

**Affiliations:** 1 Department of Pathology, School of Medicine, and Institute of Biomedical Science and Technology, Konkuk University Glocal Campus, Seoul, South Korea; 2 Department of Emergency Medicine, Konkuk University Hospital, Seoul, South Korea; 3 Department of Forensic Medicine, School of Medicine, Konkuk University, Seoul, South Korea; 4 Department of Anatomy, School of Medicine, Konkuk University, Seoul, South Korea; 5 Department of Culinary and Service Management, College of Hotel & Tourism Management, Kyung Hee University, Seoul, South Korea; 6 Department of Internal Medicine, School of Medicine, Konkuk University Glocal Campus, Chung-Ju, South Korea; 7 Department of Animal Science, College of Life Sciences, Pusan National University, Pusan, South Korea; 8 Genomic Informatics Center, Hankyong National University, Anseong, South Korea; 9 Department of Applied Plant Sciences, Kangwon National University, Chuncheon, South Korea; 10 Functional Food and Nutrition, Rural Development Administration, Suwon, South Korea; 11 Department of Applied Life Science, Konkuk University, Seoul, South Korea; King Faisal Specialist Hospital & Research Center, Saudi Arabia

## Abstract

**Background:**

Cancer is one of the highly virulent diseases known to humankind with a high mortality rate. Breast cancer is the most common cancer in women worldwide. Sorghum is a principal cereal food in many parts of the world, and is critical in folk medicine of Asia and Africa. In the present study, we analyzed the effects of HSE in metastatic breast cancer.

**Methodology/Principal Findings:**

Preliminary studies conducted on MDA-MB 231 and MCF-7 xenograft models showed tumor growth suppression by HSE. Western blotting studies conducted both *in vivo* and *in vitro* to check the effect of HSE in Jak/STAT pathways. Anti-metastatic effects of HSE were confirmed using both MDA-MB 231 and MCF-7 metastatic animal models. These studies showed that HSE can modulate Jak/STAT pathways, and it hindered the STAT5b/IGF-1R and STAT3/VEGF pathways not only by down-regulating the expression of these signal molecules and but also by preventing their phosphorylation. The expression of angiogenic factors like VEGF, VEGF-R2 and cell cycle regulators like cyclin D, cyclin E, and pRb were found down-regulated by HSE. In addition, it also targets Brk, p53, and HIF-1α for anti-cancer effects. HSE induced G1 phase arrest and migration inhibition in MDA-MB 231 cells. The metastasis of breast cancer to the lungs also found blocked by HSE in the metastatic animal model.

**Conclusions/Significance:**

Usage of HS as a dietary supplement is an inexpensive natural cancer therapy, without any side effects. We strongly recommend the use of HS as an edible therapeutic agent as it possesses tumor suppression, migration inhibition, and anti-metastatic effects on breast cancer.

## Introduction

Sorghum (Sorghum bicolor Moench) is a principal cereal food crop in many parts of the world, and the fifth most significant cereal crop in the world after wheat, rice, corn and barley [1]. Particularly, sorghum is critical in folk medicine practiced in Asia and Africa [2]. The survival of sorghum is higher than that of other cereal food crops, and is hence more economical to produce. The use of sorghum has a great potential, due to its agronomic properties, as well as emerging evidence concerning the possible beneficial biological effects of its phytochemicals. Sorghum is rich in phytochemicals known to significantly affect human health, such as tannins, phenolic acids, anthocyanins, fosters, and policosanols, which are secondary plant metabolites or integral cellular components [1]. Recent studies have proven that sorghum has anti-esophageal cancer effects [3], cholesterol-lowering effects [4], can decrease the risk of cardiovascular disease [5], and particularly showed high antioxidant activity [6]. The tannins in sorghums have the highest levels of antioxidants of any crop analyzed. Tannins of sorghum are 15∼30 fold more effective at quenching peroxyl radicals than simple phenolics [7]. Furthermore, sorghum was more cytotoxic to human cancer cells than the anthocyanidin analogues, cyanidin and pelargonidin [8]. Free radicals, chemical reactions, and several redox reactions of various compounds may produce protein oxidation, DNA damage, and lipid peroxidation in living cells [9].

Cancer is one of the highly virulent diseases known to humankind and usually results in death. Breast cancer is the most common cancer in women worldwide. Cancer has a characteristic of uncontrolled growth of abnormal cells resulting in tumor formation [8]. Tumor promotion is a reversible event during cancer development [10]. Therefore, early intervention to target inhibition of cancerous cell proliferation is very important for anti-cancer biology. An imbalance between apoptosis and cell proliferation has been implicated in breast cancer development. Apoptosis and inhibition of tumor cell proliferation have been used as markers for the evaluation of phytochemical anti-cancer activity, and many chemotherapeutic agents exerted their effects by these mechanisms [11].

The cell cycle is mainly regulated by cyclin-dependent kinases (CDKs) and cyclins. Once the cell is triggered by a mitogenic signal in early G1 phase, Cyclin D is expressed and subsequently binds with CDK4/6. This leads to the phosphorylation of Rb through Cdk-activating kinase (CAK). Activation of Rb protein causes the release of E2F, which then stimulates the expression of various cell cycle promoters [12]. The progression of G1 phase and initiation of S phase is regulated by the Cyclin/CDK complex. CDK inhibitors (CKIs) such as p21, p27 and p57, constrain the activity of the Cyclin/CDK complex. The p21 and p27 elicit G1 phase arrest either by preventing the phosphorylation of Cyclin/CDK target proteins [13], [14] or by introducing weak interaction between the components [15]. Cyclin D overexpression is observed in ∼50% of human breast cancers [16]. Specific inhibition of Cyclin D, E and over expression of CKIs can lead to suppression of tumor growth by cell cycle arrest at the G1 phase, and this provides another possibility for tumor management.

Human breast cancer cell growth, differentiation, and survival are regulated by signal transducers and activator of transcription 5 (STAT5) [17], [18]. We have reported that the Janus kinase 2 (Jak2)/STAT5b pathway regulates the transactivation of the Cyclin D1 signal pathway and insulin-like growth factor-1 (IGF-1) pathway in many solid tumor cells [19]–[23]. STAT5b is identified as an integral factor for breast cancer cell migration. STAT5b shows β1-integrin-mediated migration in the highly aggressive breast cancer cell line, MDA-MB 231 [24]. Compared with normal breast tissue, IGF-1R expression is detected at very high frequency in breast cancer specimens [25]. Many *in vitro* and *in vivo* studies provided substantial evidence that insulin-like growth factor (IGF) and the IGF-1R signaling pathway are the major regulators of growth, survival, migration and invasion in human breast cancer [26], [27]. Studies have also shown that elevated levels of serum IGF-I are correlated with increased breast cancer risk [28]. Thus, specific inhibition of STAT5b/IGF-1R could be another strategy for blocking tumor growth.

Signal transducer and activator of transcription 3 (STAT3) is an oncogenic transcription factor involved in the development and progression of various malignancies, including breast cancer [13]–[18]. We have reported that STAT3 modulates VEGF through HIF-1α [29]. Vascular endothelial growth factor (VEGF) is a regulator of angiogenesis (the process of forming new capillaries) in tumor progression [30]. VEGF expression has been reported in a number of cancer cell lines, and in several clinical specimens derived from cancerous tumors [31]. Thus, inhibition of VEGF can effectively prevent tumor growth via incomplete blood vessel formation. VEGF binds to its cognate receptor VEGF-R2. VEGF-R2 is responsible for initiating signal transduction pathways within endothelial cells. Cancer chemotherapy has gradually improved with the development of novel antitumor drugs. However, many therapeutic anticancer agents exhibit tolerance and potent cytotoxic activities against normal cells. This fact is critical if we are to progress in developing sorghum foods and food ingredients that aim to prevent and cure the onset of cancer.

In spite of a diversity of phytochemicals in sorghum, research on this crop as a source of valuable health promoting compounds lags behind research on fruits and other vegetables [1]. Sorghum is a candidate food that deserves systematic investigation. We screened HS for its anticancer activity *in vitro* and *in vivo*. We predicted that specific blocking of STAT5b in human breast cancer cells by HS may enhance solid cancer cell apoptosis. *In vitro* and *in vivo* treatment of breast cancer cells with *Hwanggeumchal sorghum* extracts (HSE) induced growth arrest and apoptosis in conjunction with the blockade of the constitutively active the Jak/STAT signaling pathways. We found that HSE can block angiogenesis by modulating the STAT3/VEGF pathway and VEGF-R2 level in human breast cancer cells. Additionally, HSE demonstrated inhibition of metastasis in MDA-MB 231 and MCF-7 induced metastatic animal models.

## Results

### HSE suppressed the growth of human breast cancer xenografts in mice

**Figure 1:**
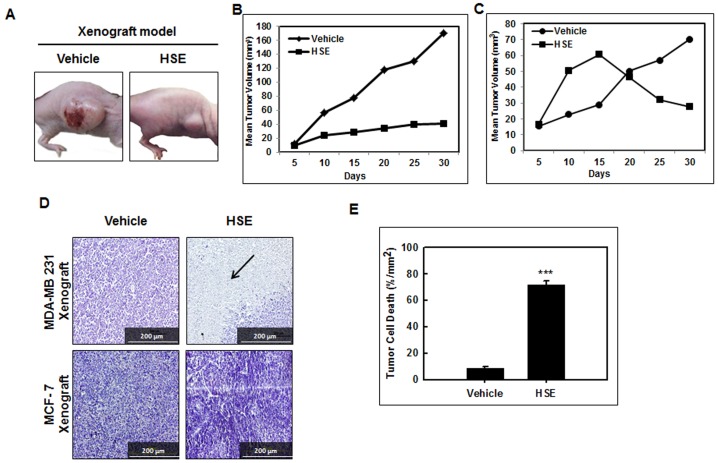
HSE suppressed the growth of human breast cancer xenograft in mice. Mice were treated daily with HSE at a dose ranging from 0 and 10 mg per kg body weight by intragastric administration. A, image of tumor-xenografted nude mice model at the end of the treatment. B, tumor size growth curves of MDA-MB 231 xenograft model during the treatment calculating the volume size of individual tumors. C, tumor size growth curves of MCF-7 xenograft model during the treatment calculating the volume size of individual tumors. D, H&E staining of MDA-MB 231 and MCF-7 xenografts. Dii, Image of necrosis in MDA-MD 231 breast tumor tissue sections at the end of the treatment. Arrow points the area of necrosis. Tumors were collected on day 30 after start of treatment and were formalin-fixed paraffin-embedded followed by staining with hematoxylin and eosin. Representative images are shown (C, magnification: ×200, scale bar = 200 µm). E, quantitative representation of tumor necrotic areas in comparison with vehicle. The area of necrosis in each section was quantified from six fields (magnification: ×200). The values are means ± S.E (n = 6) after normalization to vehicle (internal control). Asterisks indicate a statistically significant decrease (B) or increase (E) by t-test (*p<0.05, ***p<0.001).

Human breast cancer cell line MDA-MB 231 (1×10^7^ cells) was implanted into the right flank of Balb/c athymic nude mice. The mice were divided into groups of 2 and treated with HSE. For MCF-7 xenograft, 1×10^7^ MCF-7 cells were injected to the right flank of the overiectomized balb/c athymic nude mice and a 17β estradiol pellet (0.72 mg, 60 days release; Innovative Research of America, FL) was implanted into the back side of neck, to facilitate optimum growth. HSE treatment started two weeks after injection with MDA-MB 231 or MCF-7 cells. HSE was administered daily at a dose of 0 and 10 mg per kg body weight through intragastric administration. We observed a maximum inhibition of xenograft growth after two weeks of treatment with HSE in both experimental systems. The duration of treatment was four weeks. And the mice were sacrificed after six weeks. The rate of tumor growth in untreated mice (100% vehicle, n = 6) was significantly greater than that in mice treated with 10 mg HSE/kg body weight (2.3%, n = 6) (Fig. 1A, 1C). HSE treatment significantly decreased the growth of human breast tumor xenografts in mice. Furthermore, the comparison of average weekly tumor sizes generated revealed that the tumor growth in mice treated with HSE was more suppressed than in the vehicle control group in both MDA-MB 231 and MCF-7 xenograft models (Fig. 1B and C). These data suggest that HSE contributed to the inhibition of tumor growth in the xenograft animal model. All macroscopically visible mammary tumors were measured by a vernier caliper, and the tumor volume was estimated by measuring the length and width of tumors. Histological examination of the tumors was performed on sections stained with hematoxylin and eosin (H&E). As shown in Fig. 1D and E, the HSE treated group showed markedly increased tumor cell death in MDA-MB 231 model compared to the untreated vehicle group (***P<0.001). A sharp border between necrotic and viable cells was shown in the HSE treated group (Fig. 1D) whereas no notable necrosis observed in MCF-7 model. In the xenograft model, apoptosis was associated with tumor necrosis. In comparison, tumor necrosis were increased in the tumors treated with HSE, but to a lesser extent than in the vehicle tumors. These data convincingly illustrate that some tumors undergo extensive loss of viability associated with increased apoptosis after HSE treatment.

### HSE down-regulated STAT5b/IGF-1R and STAT3/VEGF signal pathways, and inhibited HIF-1α related-protein expression in human breast cancer xenografts

**Figure 2:**
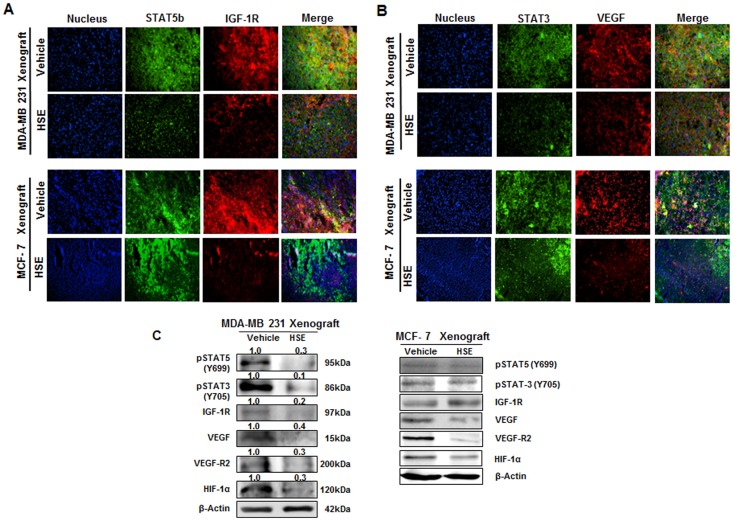
HSE down-regulated STAT5b/IGF-1R and STAT3/VEGF signal pathway, and inhibited HIF-1α related-protein expression in human breast cancer xenografts. A and B, immunohistochemical (IHC) analysis of STAT5b/IGF-1R and STAT3/VEGF protein expression in both MDA-MB 231 and MCF-7 breast tumor tissues. The xenografts were sliced to 5 µm thickness and treated with primary antibodies specific for STAT3, STAT5b, VEGF and IGF-1R. Detection was done using secondary antibody, Alexa Fluor 488 (rabbit) and Alexa Fluor 594 (mouse). The IHC staining was performed thrice (magnification: ×400). C, studies confirmed the decrease in expression of STAT5b, IGF-1R, STAT3 and VEGF with no much alteration in the nucleus level. A, IHC specific for STAT5b/IGF-1R and B, IHC specific for STAT3/VEGF. C, the expression of p-STAT5, p-STAT3, p-IGF-1R, VEGF, VEGF-R2 and HIF-1α in breast cancer xenografts. The protein extracts (10 µg) were separated by 10% SDS-PAGE, and western blots were performed as described in experimental procedures. β-actin was used as a control for protein loading.

To access the effect of HSE on the Jak2/STAT5b pathway, the levels of various proteins expressed in both breast cancer xenograft mice models were examined. We hypothesized that HSE could suppress phosphorylation of STAT5 and STAT3, and the expression or release of IGF-1R, VEGF, VEGF-R2 and HIF-1α proteins in human breast cancer xenografts *in vivo*. In the present study, the expression of STAT5b, IGF-1R, STAT3 and VEGF were analyzed using immunofluorescence microscopy. In Fig. 2A and B, HSE treatment resulted in decreased STAT5b, IGF-1R, STAT3 and VEGF expression in both MDA-MB 231 and MCF-7 xenograft models without alterations in nucleus level. As shown in Fig. 2C, HSE treatment significantly suppressed phosphorylation of STAT5 and STAT3, and the expression or release of IGF-1R and VEGF proteins. VEGF-R2 and hypoxia inducible factor 1α (HIF-1α) protein expressions were highly decreased by HSE in both xenograft models (Fig. 2C). These results are interesting because HSE was effective in inhibiting the growth and proliferation of breast cancer in general.

### HSE inhibited human breast cancer cell growth, induced G1 cell cycle arrest, and maintained the expression of tumor suppressor proteins

**Figure 3:**
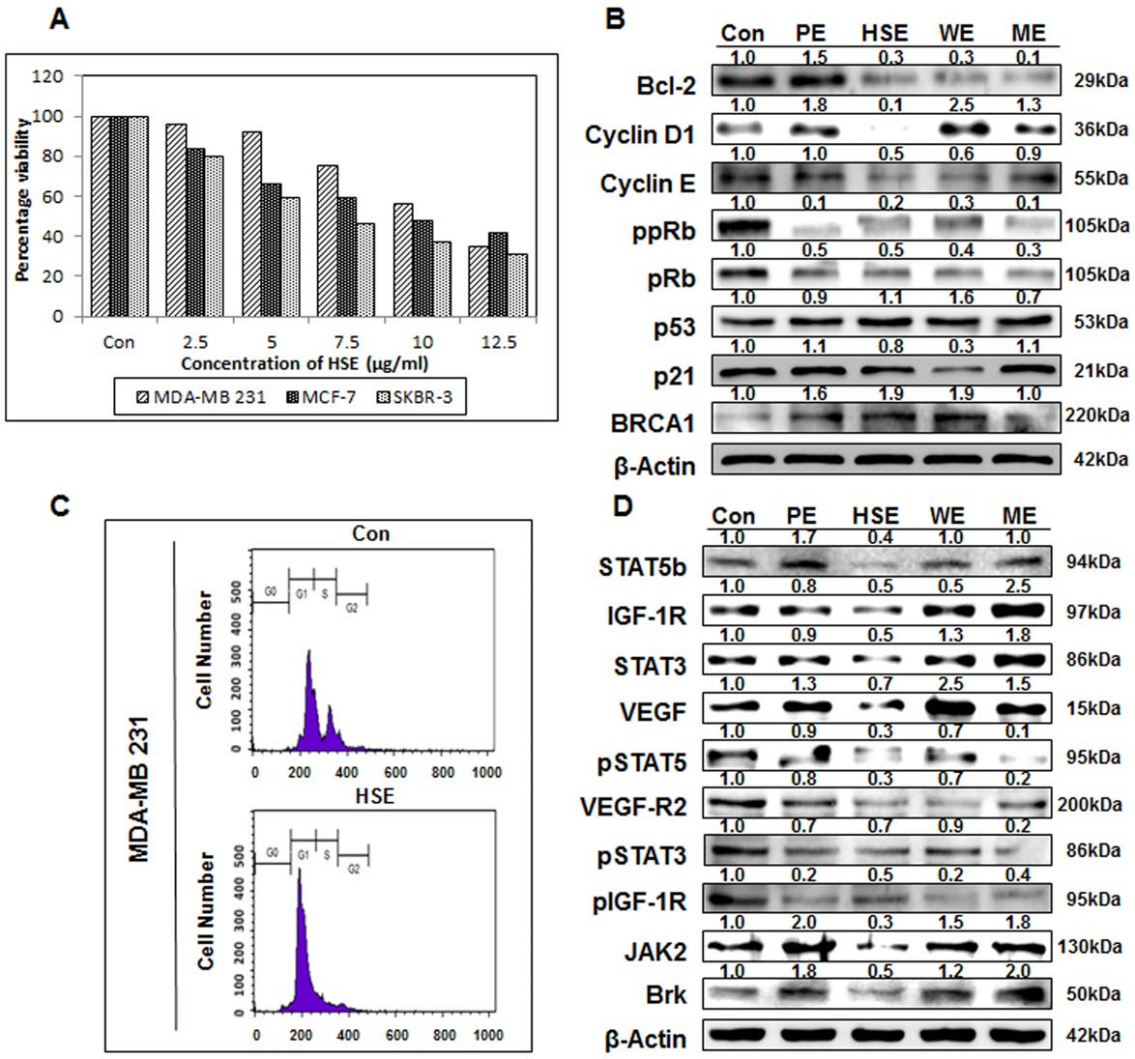
HSE inhibited breast cancer cell growth, induce G1 cell cycle arrest, maintained the expression of tumor suppressor proteins and suppressed the expression of oncogenic proteins over other whole grain extracts. A, breast cancer cells MDA-MB 231, MCF-7, and SKBR-3 were treated with HSE. After 24 h, cell viability was evaluated by the MTT assay. B, the expression of Bcl-2, Cyclin D1, Cyclin E, ppRb, pRb, p53, p21 and BRCA1 in MDA-MB 231 cells. C, the cell cycle arrest is confirmed through flow cytometry. Flow cytometry MDA-MB 231 cells using propidium iodide flow cytometry. D, the oncogenic protein expression of in MDA-MB 231 cells treated with whole grain extracts (10 µg/ml). The protein extracts were separated by 10% SDS-PAGE, and western blots were performed as described in experimental procedures. β-actin was used as a control for protein loading. Data represent the mean of at least three separate experiments, mean ± SEM. Asterisks indicate a IC_50_ value.

We investigated the anti-proliferative properties of HSE by exposing breast cancer cell lines MDA-MB 231, MCF-7, and SKBR-3 to increasing concentrations of HSE (0, 2.5, 5, 7.5, 10 and 12.5 µg/ml) for 24 h. Followed by MTT assay to assess the effect of treatment on cell proliferation. The number of HSE treated cells in the logarithmic phase of growth was compared with that of control cells. Cell growth was inhibited by ∼35% in MDA-MB 231 cells and ∼50% in SKBR-3 cells at a HSE concentration of 7.5 µg/mL. Whereas HSE inhibited ∼48% cell proliferation in MDA-MB 231 and ∼50% in MCF-7 cells at a concentration of 10 µg/ml (Fig. 3A). Thus, 10 µg/mL was concluded as the IC50. In MDA-MB 231 cells, significant G0/G1 arrest was induced after treatment with 10 µg/mL of HSE for 24 h. Approximately 46.9% of the untreated cells were in the G0/G1-phase, and this increased to 73.3% of cells after treatment with 10 µg/mL of HSE. In MDA-MB 231 cells, the G0/G1-phase fraction increased from 53.23% (untreated) to 83.12% (Fig. 3B). The expression levels of various proteins involved in the cell cycle regulation were assessed by Western blotting studies. Bcl-2 is one of the homologous proteins that had an opposing effect on cell life and death. Bcl-2 serving to prolong cell survival as an inhibitor of apoptosis. Bcl-2 expression was slightly down-regulated by the addition of 10 µg/ml of HSE (Fig. 3C). In contrast, the expression of p53 was up-regulated and p21 was maintained by HSE treatment. Cyclin D1, Cyclin E and phosphorylation of pRb are required for cell cycle progression. Changes in the Cyclin D1, Cyclin E and phosphorylation status of PRb are observed during apoptosis. Therefore, protein levels of Cyclin D1, Cyclin E and phosphorylation status of pRb in MDA-MB 231 cells were examined following treatment with HSE (10 µg/mL). MDA-MB 231 cells showed a complete inhibition of Cyclin D1 and a dramatic loss of hyper-phosphorylated forms of pRb with HSE treatment. The loss of the hyper-phosphorylated forms of pRb coincided with the induction of apoptosis.

### HSE suppressed the expression of oncogenic proteins more than other whole grain extracts

MDA-MB 231 cells were treated with 10 µg/mL of PE (Panicum extracts), HSE, WE (Wheat extracts) and ME (Millet extracts) for 24 h. As shown in Fig. 3D, the protein expression levels of STAT5b, IGF-1R, STAT3, VEGF, VEGF-R2, Jak2 and breast tumor kinase (Brk) were decreased by HSE treatment. HSE treatment also substantially suppressed phosphorylation of STAT5, STAT3 and IGF-1R proteins. These results indicate that HSE down-regulates the STAT5b/IGF-1R signal pathway and may act as a tumor growth inhibitor. In addition, HSE regulates the STAT3, VEGF and VEGF-R2 signal pathways in human breast cancer cells and thus may be an anti-angiogenic agent. Other critical factors such as p-STAT5, p-STAT3, p-IGF-1R, Jak2 and Brk causing breast cancer were also suppressed by HSE. However, BRCA1 levels were overexpressed. As a control, β-actin expression was shown to be unaffected.

### HSE down-regulated the STAT5b/IGF-1R and STAT3/VEGF signal pathways in time and dose dependent manners

**Figure 4:**
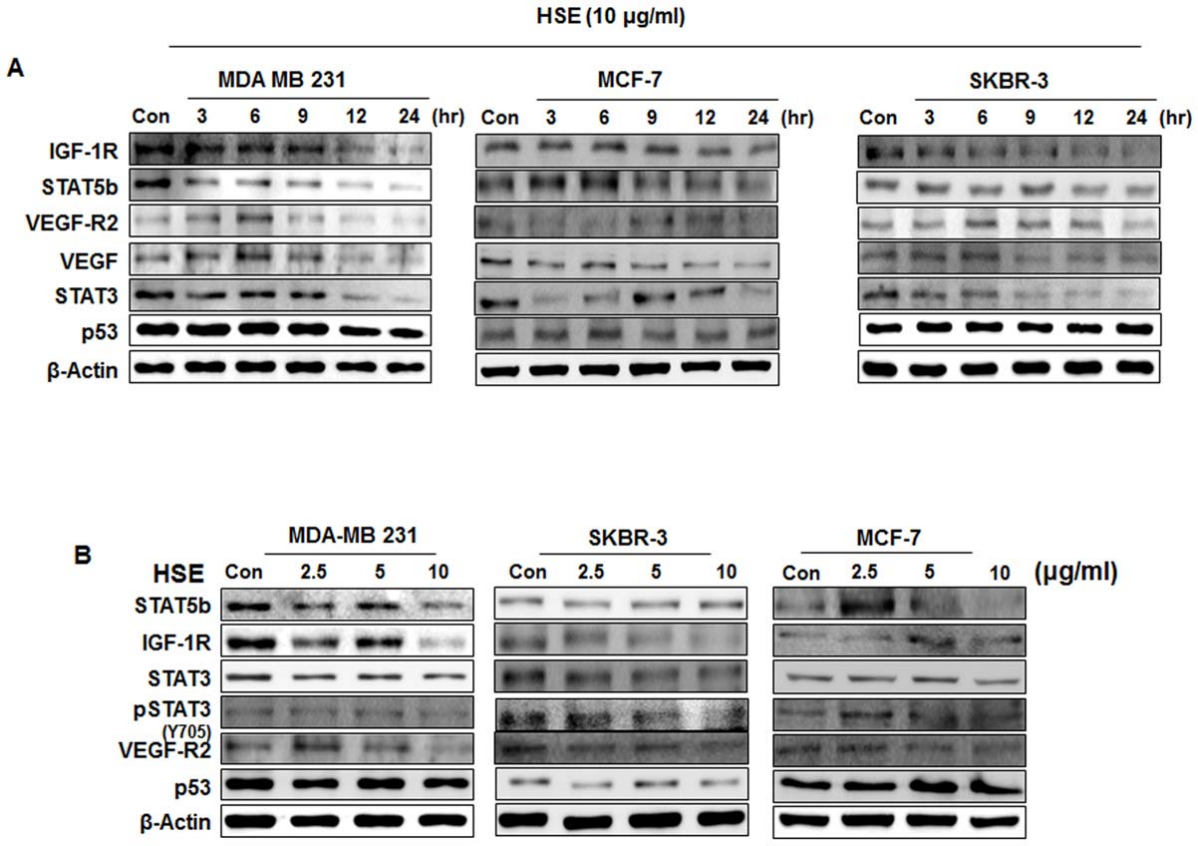
HSE down-regulated the STAT5b/IGF-1R and STAT3/VEGF pathways in time and dose dependent manner. A, human breast cancer cells MDA-MB 231, MCF-7, and SKBR-3 were exposed to different incubation time with HSE (10 µg/ml). B, MDA-MB 231, MCF-7, and SKBR-3 cells were exposed to various concentrations of HSE. The protein extracts (10 µg) were separated by 10% SDS-PAGE, and western blots were performed as described in experimental procedures. β-actin was used as a control for protein loading.

We found that HSE had a dose-dependent effect on breast cancer cell lines, MDA-MB 231, MCF-7, and SKBR-3. Our next aim was to analyze the time dependency of HSE on protein expression. Human breast cancer cell lines MDA-MB 231, MCF-7, and SKBR-3 were exposed to 10 µg/mL concentration of HSE for different time intervals (0, 3, 6, 9, 12 and 24 h). The results showed a time-dependent decrease in the protein expression of STAT5b, IGF-1R, STAT3, VEGF and VEGF-R2, with a stable expression of p53 (Fig. 4A). Even though the level of protein expression regulation varied between the cell lines, the profile of regulation was similar. HSE exhibited a maximum inhibition towards STAT5b and VEGF-R2. When these cells were treated with HSE, the protein levels of STAT5b, IGF-1R, STAT3, pSTAT3, and VEGF-R2 were markedly reduced in a dose-dependent manner. However, the levels of the p53 were maintained (Fig. 4B).

### HSE suppressed the binding activities of STAT5b to the IGF-1R and STAT3 to the VEGF promoter sites

**Figure 5:**
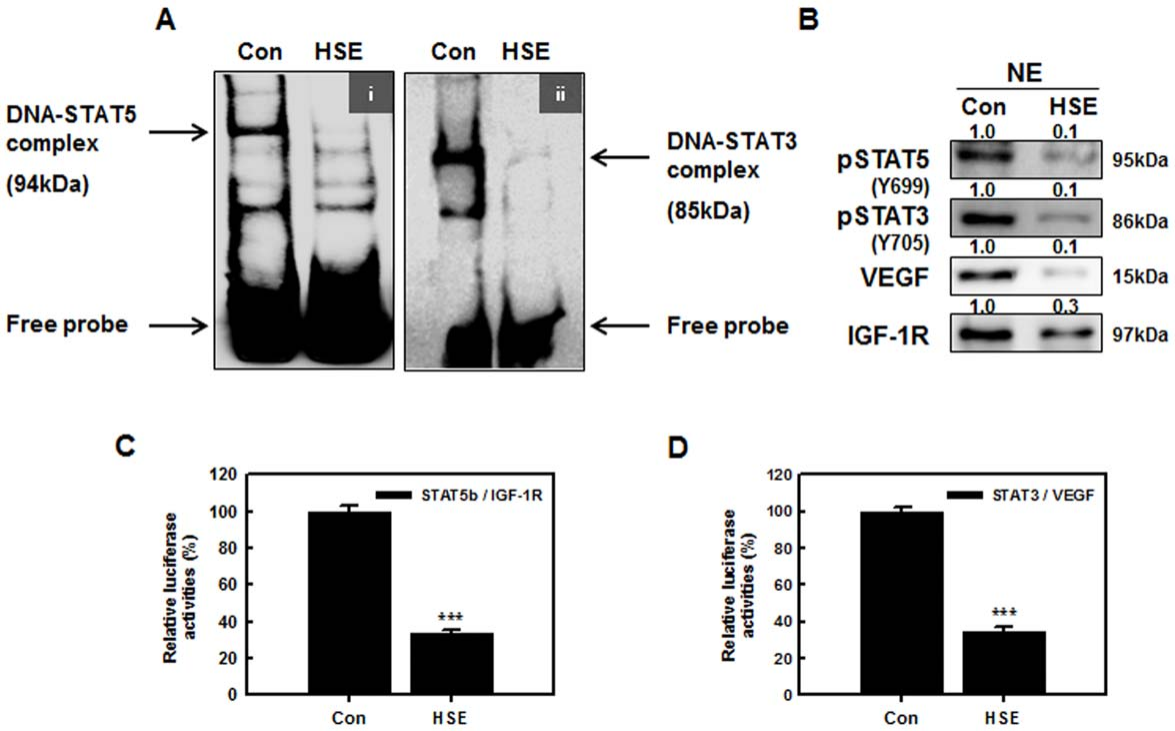
HSE suppressed the binding activities of STAT5b to the IGF-1R and STAT3 to the VEGF promoter sites. A, electrophoretic mobility shift assay (EMSA) showed, HSE suppressed the DNA-binding activity of STAT5b to the IGF-1R (i) and STAT3 to the VEGF (ii) binding sites in MDA-MB 231 cells treated with HSE for 24 h. B, nuclear protein extracts (NE) were separated and blotted onto nitrocellulose membrane showing decrease in the level of p-STAT5, p-STAT3, VEGF and IGF-1R. C and D, shows the activation of STAT5b/IGF-1R promoter and STAT3/VEGF promoter in COS-7 cells by HSE. COS-7 cells were transiently co-transfected with STAT5b and IGF-1R (C) and STAT3 and VEGF genes (D). Data represent means of at least three separate experiments. Asterisks indicate a statistically significant decrease by t-test (***p<0.001) in the crosstalks of STAT5b/IGF-1R and STAT3/VEGF respectively.

Translocation of initiated STATs to the nucleus forms dimers and binds to specific response elements in the promoters of target genes, and transcriptionally activates these genes [28]. The STAT nuclear translocation and DNA binding activity are probably not influenced by STAT serine phosphorylation, but they are influenced by STAT tyrosine phosphorylation [29]. STAT5 participates in oncogenesis by up-regulating genes encoding apoptosis inhibitors and cell cycle regulators. To test the hypothesis that HSE inhibits STAT5b and STAT3, we examined the binding activities of STAT5 to the IGF-1R and STAT3 to the VEGF promoters. As shown in Fig. 5Ai and Aii DNA binding activities of STAT5b to the IGF-1R and STAT3 to the VEGF promoter were studied in HSE treated (10 µg/mL) and untreated (control) MDA-MB 231 cells. The results show that HSE suppressed STAT5 DNA-binding activity in human breast cancer cells, and there was a dramatic loss of STAT3 binding to the promoter site in HSE treated MDA-MB 231 cells. The nuclear extracts showed decreased levels of p-STAT5, p-STAT3, VEGF and IGF-1R (Fig. 5B). Hence, HSE inhibited the phosphorylation of STAT5b to p-STAT5, and binding to the promoter site of IGF-1R. Furthermore, HSE inhibited the phosphorylation of STAT3 to p-STAT3, and binding to the promoter site of VEGF. In addition, HSE treatment inhibited STAT5b/IGF-1R and STAT3/VEGF-dependent luciferase activities in breast cancer cells. We performed luciferase reporter assays to determine the effect of HSE treatment on transcriptional activities of STAT5b/IGF-1R and STAT3/VEGF genes (Fig. 5C and D). As shown in Fig. 5C and D, the relative luciferase activity of STAT5b/IGF-1R and STAT3/VEGF promoters were significantly inhibited after 24 h of HSE treatment (by 65% of control, ***P<0.001). As compared with control, inhibition of the crosstalk between STAT5b and IGF-1R and between STAT3 and VEGF genes by HSE were similar. These results suggest that HSE may be a critical inhibitor of the STAT5/IGF-1R and STAT3/VEGF pathways.

### Suppression of STAT3/DNA binding activity leads to the crackdown of target gene products

**Figure 6:**
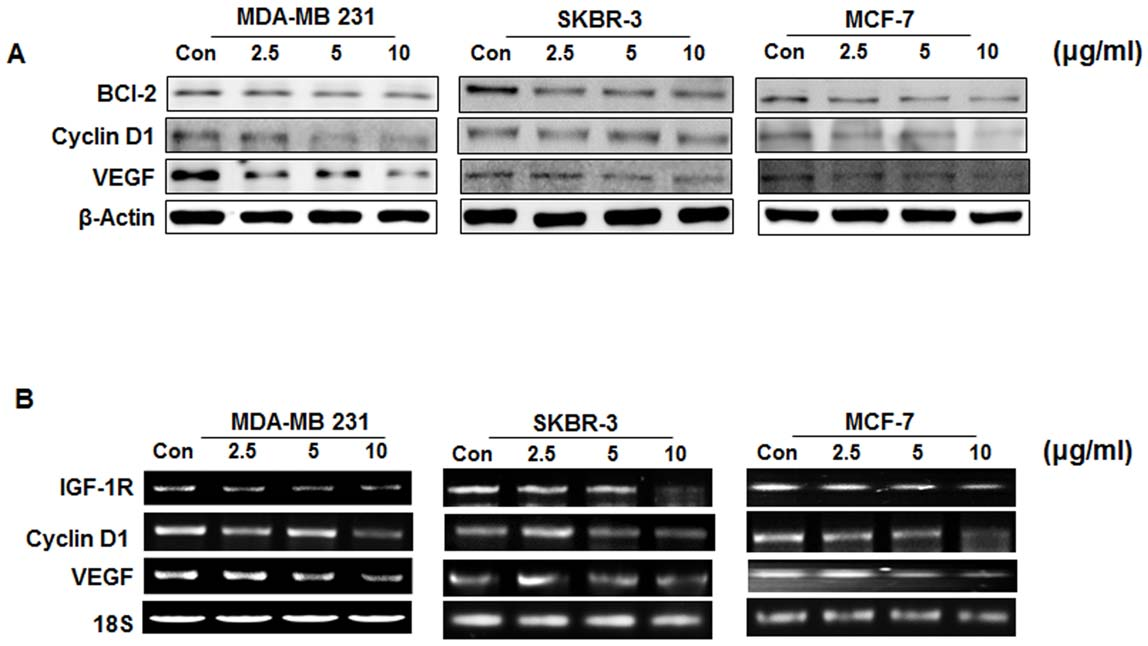
Suppression of STAT3/DNA binding activity leads to the crackdown of target gene products. A, western blotting analysis of whole cell lysates from MDA MB-231, MCF-7 and SKBR-3 cells treated with increasing concentration of HSE. B, RT-PCR analysis of MDA-MB 231, MCF-7, and SKBR-3 cells treated with different concentration of HSE showed transcriptional regulation of IGF-1R, Cyclin D1 and VEGF mRNA levels. 18S RNA was used as a control.

As expected from the crackdown of STAT3 and pSTAT3 abundance and DNA binding activity, HSE exposure led to the down-regulation of STAT3 target gene products, such as VEGF, BCl-2, and Cyclin D1 (Fig. 6A). By modulating these molecules, HSE can target angiogenesis and metastasis (VEGF), cell cycle (Cyclin D1) and anti-apoptosis (BCl-2).

RT-PCR studies were carried out in these cells treated with increasing concentrations of HSE by random priming of total RNA. IGF-1R, Cyclin D1 and VEGF were amplified using gene specific primers. RT-PCR on both treated, and untreated HSE yielded amplified products of IGF-1R, Cyclin D1, and VEGF mRNA (Fig. 6B). As shown in Fig. 6B, HSE down regulated the transcription of STAT3 targets in a dose-dependent manner. 18S was used as a control for DNA loading. As a control, 18S expression was shown to be unaffected following; IGF-1R, Cyclin D1 and VEGF were suppressed at the RNA level. Expression of the VEGF gene is affected by interactive agents in various human breast cancer cell lines [24]. In a growing tumor mass, VEGF expression is physiologically caused by many types of tumors and in regions surrounding necrosis [25], [26]. The abnormal formation of new blood vessels requires the involvement of VEGF, VEGF-R2 and IGF-1R [27]. Thus, IGF-1R, VEGF and its receptors are considered as potential targets for therapeutic intervention. These results clearly suggest that HSE could be useful for the treatment of breast cancer.

### HSE might block the metastasis of breast cancer to lungs in an animal model

**Figure 7:**
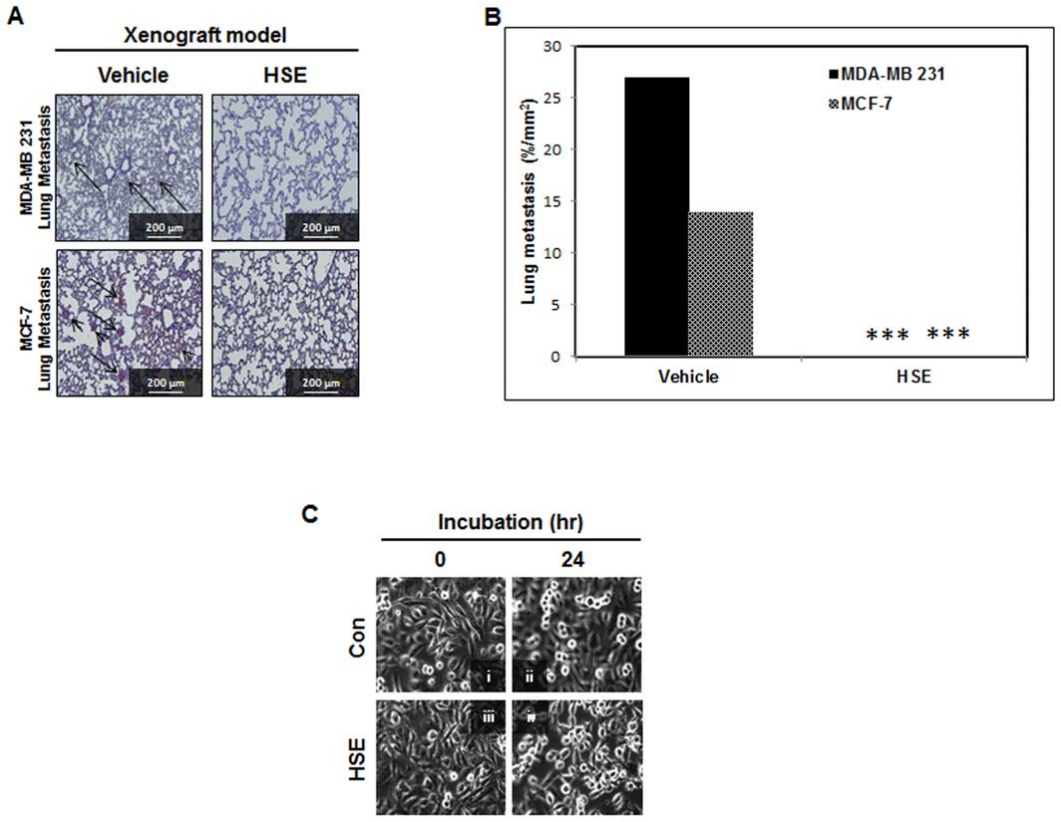
HSE might block the metastasis of breast cancer to lungs in animal model. A, analysis of the effect of HSE on the experimental lung metastases established by MDA-MB 231 and MCF-7 cells. Mice were injected with MDA-MB 231 cells (1×10^7^ in 200 µL PBS per mouse) and MCF-7 cells (1×10^7^ in 200 µL PBS per mouse). They are randomized into two groups for each cell model. One group of mice (n = 6) was injected with HSE for 30 days, and the other group of mice (n = 6) was injected with water using the same schedule. Lungs were collected on day 30 after the start of treatment and formalin-fixed paraffin-embedded lung tissues were stained with hematoxylin and eosin. Representative images are shown (A, magnification: ×200, scale bar = 200 µm). Arrow points the area of metastasis. B, quantitative analysis of tumor areas shown in A. The area of metastatic nodules in each section was quantified from six fields (magnification: ×200). The values are means ± S.E (n = 6) after normalization to vehicle (internal control). Asterisks indicate a statistically significant decrease by t-test (***p<0.001). C, HSE inhibited the migration of MDA-MB 231 cells treated with HSE (10 µg/ml for 24 h). MDA-MB 231 cells were plated into DMEM medium separately. After 24 hours, medium was replaced with and without HSE (10 µg/ml). Live cell images were acquired 24 hours after media changes (i and ii; pre-confluent), (iii and iv; confluent). Movie S1, MDA-MB 231 cells without HSE (control) and Movie S2, MDA-MB 231 cells treated by HSE.

From *in vitro* analysis, it was clear that HSE inhibited STAT3, VEGF and VEGF-R2 in the dose and time dependent manners. This provides a direct indication that HSE can inhibit tumor angiogenesis and thereby prevent metastasis. To confirm this, we examined whether HSE could cause regression of established experimental lung metastases induced with MDA-MB 231 and MCF-7 cells in Balb/c athymic nude mice after treatment with HSE (10 mg/kg of body weight) for a period of four weeks; a time by which vehicle treated MDA-MB 231 and MCF-7 cell models showed measurable levels of lung metastasis. The lungs were removed from mice and areas of lung metastases were quantified. As we expected, metastatic locus was detectable in histologic sections from mice injected with cells (Fig. 7A). In animals treated with vehicle, higher levels of metastasis were observed with increased numbers of metastatic loci and increased total metastatic area. However, no metastases were observed in HSE treated metastatic animal models (Fig. 7B, ***P<0.001). Live cell video microscopy demonstrated that metastatic breast cancer cells in 10 µg/mL HSE reproduced and migrated until the cells came in contact with neighboring cells. At this point, breast cancer cells cultured in the absence of HSE retained an amorphous worm-like shape, and after reaching confluence, continued to migrate under and over adjacent cells (Fig. 7C; Movie S1). In contrast, cells in HSE stopped migration and formed a confluent monolayer of contact inhibited cells (Fig. 7C; Movie S2). The anti-angiogenic effect was produced by modulating STAT3/VEGF and VEGF-R2, contact inhibition and migration inhibition together constituted the anti-metastasis effect of HSE.

## Discussion

Targeted therapy for treating cancer is a new approach to reduce maximum side effects and provide maximum cure rates. Because of the complex signaling pathways present in the cancer cell, successful targeted therapies are becoming more difficult to identify. Each cellular process is controlled by various signal networks and blocking a single signal pathway stimulates the cell to bypass the mechanism and produce and find another means to achieve the same cellular effect. This results in the need for multiple targeted therapies. We found that HSE (*Hwanggeumchal sorghum* extracts) is able to inhibit and regulate multiple signaling points. HSE directly shows its ability to control various cellular processes such as cell cycle by inhibiting the expression of Cyclin D1, tumor growth and metastasis by down-regulating the Jak2/STAT pathways. STAT3 is involved in tumor progression by inducing angiogenic factors such as VEGF [33]. HSE also has a regulatory effect in tumor angiogenesis by suppressing VEGF and VEGF-R2. VEGF and particularly VEGF-A, is considered to be the most important and potent pro-angiogenic factor involved in tumor growth [30], and VEGF-R2 is the major receptor associated with tumor angiogenesis [34].

Sorghum is rich in secondary plant metabolites or integral cellular components such as tannins, phenolic acids, anthocyanins, phytosterols and policosanols, which are known to significantly affect human health [1]. Recent studies have proved that sorghum has anti-oesophageal cancer effects [3], cholesterol-lowering effects [4], and can decrease the risk of cardiovascular disease [5], particularly showed high antioxidant activity [6]. We needed to check the effect of sorghum in breast cancer management. For detecting its ability for tumor growth suppression, tumor xenografts were established in Balb/c athymic nude mice by implanting the MDA-MB 231 and MCF-7 metastatic human breast cancer cell lines into the right flank. The results obtained after administering HSE were promising. There was a mean reduction of 97.7% in tumor volume (Fig. 1A, B, & C). H&E staining of these xenografts showed a marked decrease of viable cells in the tissue (Fig. 1Dii). HSE-treated xenografts showed markedly increased tumor cell death compared to controls, and there was a mean reduction of 65.2% in cell death (***P<0.001; Fig. 1E). These finding suggest the potential of HSE in breast cancer management.

Tumor growth is angiogenesis dependent and VEGF and VEGF-R2 are the prime requirements for angiogenesis [35]. Previous studies showed that the level of VEGF secretion can directly influence tumor size. In most tumors, the size is proportional to VEGF secretion and larger tumors have increased VEGF levels [36]. Our studies using breast tumor xenografts also showed decreased expression of VEGF and VEGF-R2 (Fig. 2B and C); therefore, we assume that HSE has an effect on inhibiting tumor angiogenesis. Tumor angiogenesis is not only dependent on VEGF or VEGF-R2. Hypoxia is a major factor stimulating angiogenesis, resulting in increased levels of hypoxia inducible factor -1 (HIF-1). HIF-1α induces the release of various angiogenic factors including VEGF [37]. Regulation of HIF-1α is also required for effective regulation of tumor angiogenesis and tumor growth suppression. HSE showed a good control of HIF-1α expression Fig. 2C).

Activation of the IGF-I receptor plays an important role in cellular transformation, mitogenesis, inhibition of apoptosis, proteolytic degradation of extracellular matrix and tumor angiogenesis [38]. Overexpression of IGF-1R is observed in all types of solid tumors [39]. In most tumors this IGF autocrine group with HIF-1 emerges as a survival strategy [40]. In many tissues and tumor types, the Jak/STAT pathway has been implicated as an important mediator of IGF dependent signaling pathways [41], [42]. Therefore, a molecule that inhibits IGF-1R should block the IGF autocrine loop as well as the Jak/STAT signaling. HSE showed a down-regulation in IGF-1R (Fig. 2A) and suppressed the activation of IGF-1R (Fig. 2C). Additionally, the expression and activation of STAT3 (Fig. 2B and C) and STAT5b (Fig. 2A and C) were found to be down-regulated by HSE treatment. This clearly indicates that HSE possesses apoptotic, anti-metastatic and anti-cancer effects.

The anti-cancer effect and IC_50_ of HSE were detected *in vitro*, using human breast cancer cell lines MDA-MB 231, MCF-7, and SKBR-3. The IC_50_ was determined to be 10 µg/mL (Fig. 3A). Our study showed that treatment with HSE arrested the growth of metastatic human breast cancer cells in the G1 phase (Fig. 3C). The progression of cell cycle from G1 to S phase requires the activation of CDK4 and CDK2 by Cyclin D and Cyclin E [43], [44]. HSE demonstrated a near complete inhibition of Cyclin D1and Cyclin E in MDA-MB 231 cells (Fig. 3B) and this could be the reason for growth arrest. Comparative analyses of other whole grain extracts with HSE also showed its potential for effective cell cycle regulation (Fig. 3C). HSE also down-regulated the expression and phosphorylation of Rb protein (Fig. 3B). In G1 phase, phosphorylation of Rb protein causes the activation and release of E2F. This E2F activates the expression of S phase genes [45]. Hypophosphorylated Rb produced by the action of HSE might result in decreased release of E2F or increased interaction of E2F with hypophosphorylated Rb, resulting in G1 arrest. p21 expression may be regulated either in p53 dependent or independent manners [46]–[48]. In our study, an increase in the expression of p53 as well as p21 was found, and there is a possibility that induction of p21 by HSE is related to the regulation of p53.

The anti-tumor and anti-metastatic effects of HSE found in the *in vivo* study were also analyzed *in vitro*. A comparative analysis of other whole grain extracts with HSE was also conducted to identify promising anti-cancer agents. HSE showed a better anti-cancer effect by demonstrating high efficacy in down-regulation of signal molecules (Fig. 3D).

STAT5b is a transcription factor involved in the proliferation and survival of many solid tumors, including breast cancer [24], [42]. The activation of STAT5b and phosphorylation is mediated by several kinases overexpressed in breast cancer. Protein tyrosine kinase 6 (PTK6), also known as breast tumor kinase (Brk), is a non-receptor tyrosine kinase overexpressed in almost all type of breast cancers [49]. Very few substrates of Brk tyrosine kinase have been identified, which includes STAT5b and STAT3 [50], [51]. HSE effectively down-regulated the expression of Brk in breast cancer cell lines by the activation of STAT3 and STAT5b (Fig. 3D). Janus kinase-2 (Jak2) is another kinase enzyme involved in the activation and phosphorylation of STAT molecules. Jak2 is a new target for tumor growth suppression due to its role in the activation of STAT3 [52]. HSE suppressed the expression of Jak2 in metastatic breast cancer cell line MDA-MB 231 (Fig. 3D). Suppression of Jak2 expression may also influence the hypophosphorylation of STATs. Another possible factor behind the hypophosphorylation of STAT3 is suppressed phosphorylation of IGF-1R by HSE.

HSE not only suppressed the activation of STAT3 and STAT5b, but also their expression (Fig. 3D). HSE demonstrated a dose and time dependent suppression on the expression of STAT5b and STAT3 (Fig. 4A and B). STAT5b is involved in the β1-integrin mediated migration of breast cancer cells. Phosphorylation and activation of STAT5b is mainly dependent on the phosphorylation of tyrosine 699 (Y699). However, the migratory function of STAT5b does not require this phosphorylation at Y699 or the C-terminal transactivation domain [24]. The ability of HSE to regulate the expression level of STAT5b may contribute to anti-migratory and anti-metastatic effects. In our present study, we found that HSE inhibited cellular migration of the MDA-MB 231 metastatic breast cancer cell line (Fig. 7C, Movie S1 & S2). STAT5b usually binds with the promoter sites of IGF-1 and promotes its transcription. Therefore decreased expression of STAT5b may result in decreased transcription of IGF-1. HSE showed a decrease in the binding of STAT5b to the IGF-1R promoter (***P<0.001; Fig. 5Ai and C). This decrease in the DNA binding and promoter activities cracked down the expression of down-stream target molecule like BCl-2, Cyclin D1 and VEGF (Fig. 6). In addition to this HSE showed time and dose dependent inhibitory effect on IGF-1R (Fig. 4, 6B). IGF-1R has an important role in anchorage independent growth of cells, leading to metastasis of cancer [53]. Another important finding about IGF-1R is the activation of proto-oncogenes, and loss of suppressor oncogenes are related to IGF-1R function [54]–[56]. Targeting IGF-1R in cancer management is a new strategy. Approaches leading to overall suppression of IGF-1R are needed rather than the agents blocking its thymidine kinase activity for providing better anti-cancer efficacies [57]. HSE was found to efficiently down-regulate the expression of IGF-1R at both the transcriptional and translational levels (Fig. 4 and Fig. 6B). Blocking of IGF-1R may inhibit tumorigenesis, induce apoptosis and inhibit tumor invasion and metastasis.

HSE exhibited inhibition of VEGF transcription (Fig. 6A). STAT3 is a factor regulating the transcription of VEGF by binding to the promoter of the VEGF gene. Constitutively active STAT3 can up-regulate VEGF transcription leading to tumor angiogenesis [58]. HSE inhibited the binding of STAT3 to VEGF (***P<0.001; Fig. 5Aii and D). In addition to this, expression of VEGF and its receptor VEGF-R2 are down-regulated in dose and time dependent manners by HSE (Fig. 4). The role of HSE in the inhibition of metastasis was confirmed *in vivo* through both MDA-MB 231 and MCF-7 cells induced experimental models. We found that HSE can inhibit the metastasis of breast cancer to the lungs (Fig. 7A and B).

We confirmed that HSE demonstrates tumor suppression, migration inhibition and anti-metastatic effects on human breast cancer cells, and the xenografts induced by MDA-MB 231 and MCF-7 cells. Through *in vitro* and *in vivo* analysis, we found Jak/STAT pathways were the major targets of HSE. Apart from that, it also targets Brk, p53, Cyclin D, Cyclin E and pRb for its anti-cancer effects. Usage of *Hwanggeumchal sorghum* as a dietary supplement may be an inexpensive natural cancer therapy, without any side effects. Therefore, we strongly recommend the use of *Hwanggeumchal sorghum* as an edible therapeutic agent.

## Materials and Methods

### Ethics statement

All procedures for animal experiment were approved by the Committee on the Use and Care on Animals (Certificate No: KUB00313, Institutional Animal Care and Use Committee, Seoul, Korea) and performed in accordance with the institution guidelines.

### Materials

ACN (acetonitrile), 0.1N HCl, Dulbecco's modified eagle's medium (DMEM), RPMI-1640, 10% fetal bovine serum (FBS) and trypsin-EDTA were purchased from Gibco-BRL (Grand Island, NY). 17β-estradiol pellets (0.72 mg, 60 days release) were purchased from Innovative Research of America, FL. Anti-actin antibody, insulin and EGF were obtained from Sigma Chemical (St. Louis, MO). Phospho-STAT3, 5, STAT3, 5b, Phospho-IGF-1R, IGF-1R, VEGF, VEGF-R2, HIF-1α, Bcl-2, Cyclin D1, E, Phospho-pRb, pRb, BRCA1, Jak2, Brk and β-actin antibodies were purchased from Santa Cruz Biotechnology, The secondary antibody (goat anti-mouse IgG-horseradish peroxidase) were obtained from Santa Cruz Biotechnology (Santa Cruz, CA). The enhanced chemiluminescence (ECL) detection kit. RT-PCR Premix kits and VEGF, IGF-1R, 18s primer for RT-PCR were synthesized by Biomeer (Daejeon, Korea). Luciferase assay (LUC assay) cell lysis and substract were purchased from Promega Corp. (Madison, WI). Restore TM Western Blot Stripping Buffer and NE-PER kit were purchased from Pierce (Rockford, IL). FuGene 6 transfection reagent was from Roche (Basel, Switzerland), RNeasy mini kit and Qiaprep spin miniprep kit were purchased from Qiagen (Germany). FuGene 6 transfection reagent was from Roche Corp (Basel, Switzerland). The electrophoretic mobility shift assay (EMSA) kit and oligonucleotide probes (STAT5) were purchased from Pamomics (Redwood City, CA). Paraformaldehyde and mounting solution in immunohistochemistry (IHC) were purchased from Dae Jung Chemicals & Metals CO. (Shiheung-city, Korea) and Life science (Mukilteo, Washington). Triton 100× were obtained from Sigma Chemical Co. (St. Louis, MO).

### Extraction and characterization of sorghum

For the present study *Hwanggeumchal sorghum* was selected. The HS was extracted by using Chung and Kim's [32] method with slight modifications. Sample (50 g) was kept at −36°C and ground well prior to use. Ground powder was then extracted with 30% 0.1N HCl in acetonitrile. The solution was filtered (whatman No. 42) concentrated and again extracted with 100% methanol and dried overnight in a Freeze dryer. This Sorghum extract is characterized for its phenolic content using HPLC (Data not shown^*^).

### Tumorigenicity

All procedures for animal experiments were approved by the Committee on the Use and Care on Animals (Institutional Animal Care and Use Committee, Seoul, Korea) and performed in accordance with the institution guidelines. MDA-MB 231 (No: 30026, KCLB, Korea) tumor xenografts were established by subcutaneously inoculating 1×10^7^ cells into the right flank of 5-week-old BALB/c nude mice (Orient Bio, Seongnam-Si, Korea). For the establishment of MCF-7 xenograft, the mice were ovariectomized. A 17β-estradiol pellet (0.72 mg, 60 days release; Innovative Research of America, Sarasota, FL) was implanted subcutaneously into the neck to facilitate optimal tumor growth for the estrogen receptor–positive MCF-7 cells. The xenografts were initiated by subcutaneously injecting 1×10^7^ MCF-7 cells into the flank of the right hind leg. When tumors reached between 6 to 8 mm in diameter, mice were randomly assigned to control group, and 10 mg per kg body weight HSE treated group, respectively with 6 mice in each group. The drug was administered as intragastric injections of 100 µl, containing 10 mg per kg body weight HSE in triple distilled water. The injections were repeated one time every other day. Tumor growth was monitored by periodic measurements with calipers. Tumor volume was calculated using the formulae:
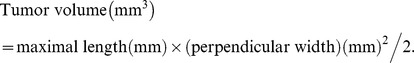
Animals were sacrificed when the diameter of tumors reached 2 cm or after 30 days of treatment. In our experiments, no mice were observed to be died of tumor loading. All available breast cancer specimens collected from in human breast cancer xenograft mice were reviewed and included in the study. Mice were euthanized and the tumors were removed. The tumors were fixed with 4% paraformaldehyde followed by paraffin embedding and sectioning (5 µm). The sections were stained with haematoxylin and eosin (H&E).

### Immunohistochemistry

Formalin-fixed paraffin-embedded xenografts of both MDA-MB 231 and MCF-7 models were sliced into 5 µm thick tissue sections. This tissue sections were deparaffinized with xylene (100%), rehydrated with decreasing concentrations (100%, 90%, 80% and 70%) of ethyl alcohol, permeabilized with triton X-100 (0.1%) and blocked with NGS (Nomal Goat Serum in PBS 10%). It is then incubated in a closed humid chamber with the STAT5b, IGF-1R, STAT3 and VEFG anti-body followed by secondary antibody, Alexa Fluor 488 (rabbit) and Alexa Fluor 594 (mouse) (Invitrogen). For nuclear staining, tissue sections were incubated with DAPI for 1 min, and rinsed with PBS. The slides were then observed under fluorescent microscope.

### Cell culture

MDA-MB 231, human breast cancer cells and COS-7 (No: 30022, KCLB, Korea), monkey kidney cells were grown to confluency in DMEM containing 10% FBS, 2 mM glutamine and 100 U/ml penicillin and streptomycin at 37°C in 5% CO_2_. MCF-7 and SKBR-3 human breast cancer cells were grown to confluency in RPMI-1640 media containing 10% FBS, 2 mM glutamine and 100 U/ml penicillin and streptomycin at 37°C in 5% CO_2_. At the initiation of each experiment, the cells were resuspended in the medium at a density of 2.5×10^5^cells/ml. The cells were placed in airtight chambers (Nu Aire, Plymouth, MN).

### Total cell lysis and western blot

The breast cancer cell lines were treated with HSE for determined times. Cells were lysed on ice with radioimmunoprecipitation assay (RIPA) lysis buffer, containing protease and phosphatase inhibitors. Cells were disrupted by aspiration through a 23-gauge needle, and centrifuged to remove cellular debris. Protein concentrations were measured using the Bradford method. Equal amounts of protein obtained by total lysis were separated by 10% SDS-PAGE and transferred to a nitrocellulose membrane. The membranes were probed with primary antibodies followed by HRP conjugated secondary antibodies. Antibody detection was done by using enhanced chemiluminescence (ECL) plus detection kit.

### Cell proliferation study by 3-(4,5-dimethylthiazol-2-yl)-2,5-diphenyl-2H-tetrazolium bromide (MTT) assay

Cell viability was assayed by measuring blue formazan that was metabolized from 3-(4,5-dimethylthiazol-2-yl)-2,5-diphenyl tetrazolium bromide (MTT) by mitochondrial dehydrogenase, which is active only in live cells. The cells were resuspended in the medium at a density of 3×10^3^cells per well in 96-well culture plates prior to each experiment. Liquid medium was replaced with a fresh medium containing DMSO (vehicle control). Cells were incubated for 24 h with various concentrations of HSE. MTT (5 mg/ml) was added to each well and incubated for 4 h at 37°C. The formazan product was dissolved by adding 200 µl dimethylsulfoxide (DMSO) to each well, and the absorbance was measured at 550 nm on Ultra Multifunctional Microplate Reader (TECAN, Durham, NC). All measurements were performed in triplicate, and each experiment was repeated at least three times.

### Reverse transcription polymerase chain reaction (RT-PCR)

Total RNA was isolated from the cells by using Tri reagent (Sigma Chemical Co., St. Louis, MO) and quantitated spectrophotometrically at 260 nm. RT-PCR analysis for VEGF, IGF-1R and 18s RNA was performed. Briefly, 4 ng of RNA was reverse transcribed, and nested PCR was carried out by using 2 µl of cDNA. The PCR condition consisted of denaturation for 1 min at 94°C, annealing for 1 min at 58°C, and extension for 1 min at 72°C. RT-PCR products were analyzed on 1% agarose gel stained by ethidium bromide.

### Cell cycle analysis

Approximately 5×10^5^ cells were separated, washed twice with phosphate buffered saline (PBS) and fixed in cold ethanol and then incubated with propidium iodide (PI) for 30 min. Thereafter, cells were analyzed by a flow cytometer (BD, NJ).

### Electrophoretic mobility shift assay (EMSA)

STAT5 and STAT3 DNA binding activity was detected using an electrophoretic mobility shift assay (EMSA), in which a labeled double-stranded DNA sequence were used as a DNA probe to bind active STAT5b and STAT3 protein in nuclear extracts. Nuclear protein extracts were prepared with the Nuclear Extract Kit (Panomics, AY2002). EMSA experiment is performed by incubating a biotin-labeled transcription factor (TF-STAT5 and STAT3) probe with treated and untreated nuclear extracts. STAT5b and STAT3 DNA binding activity was detected using an electrophoretic mobility shift assay (EMSA), in which a labeled double-stranded DNA sequence were used as DNA probes to bind with active STAT5b and STAT3 protein in nuclear extracts. EMSA was performed under the following conditions: MDA-MB 231 cells were grown to ∼90% confluence. Nuclear protein extracts were prepared with the Nuclear Extract Kit (Panomics, AY2002). Reactions were resolved on a nondenaturing 4% to 20% PAGE gel (Bio-Rad, Korea), and mobility shifts were detected by autoradiography. The proteins gel is transferred to a nylon membrane and detected using strepatvidin-HRP and a chemiluminescent substrate. The shifted bands corresponding to the protein/DNA complexes visualized were related to the unbound dsDNA.

### Co-transfection and luciferase reporter assay

The expression vectors for mouse STAT5b (pMX/STAT5b; kindly provided by Dr. Koichi Ikuta, Kyoto University, Japan) were constructed as previously described. cDNA for STAT5b was inserted into the *EcoR*I and *Sal*I sites of the pMX vector. IGF-1R (kindly provided by Dr. Haim Werner, Tel Aviv University, Israel) genomic DNA fragments including nucleotides −2350 to +640 (nucleotide 1 corresponds to the transcription start site of the rat IGF-1R gene), was subcloned upstream of a promoterless firefly luciferase reporter in the pGL2P vector (Promega, Madison, WI). And cells were co-transfected with various combinations the following constructs; wild-STAT3 (gifts from Dr. Shong, Chungnam National University, Korea) and the VEGF reporter construct containing 2.7 kb of the VEGF promoter region. COS-7 cells were cultured into 35 mm culture dishes and transfected with STAT5b or STAT3 DNA constructs (1 µg) using the FuGene 6 according to the manufacturer's recommendation. For transient cotransfection experiments, genomic DNA fragments including nucleotides IGF-1R or VEGF was subcloned upstream of a promoterless firefly luciferase reporter in the pGL2 vector (Promega, Madison, WI). Transfected cells were washed twice with ice-cold PBS, and lysis buffer was added to the wells. Lysates were then used directly to measure luciferase activity. Luciferase activity of each sample was determined by measuring luminescence for 10 sec on a Lumat LB 9507 luminometer (EG&G Berthold, Oak Ridge, TN) after injection of 100 µl of 1 mM luciferin.

### Live cell microscopy

Cells were plated on 35 mm tissue culture dishes at a concentration of 10^5^ cells/plate, and incubated at 37°C, 5% CO_2_. Time series (24 h) of phase contrast images were acquired at a video rate of 1 frame/h with a Real-time cell observer (Carl Zeiss), via the stream acquisition option of Metamorph image acquisition. During recordings, cells were kept at 37°C with pH 7.4. Time series of cell images with and without HSE were obtained after the addition of HSE, at 0 h up to 24 h incubation.

### Metastatic animal model

Primary tumors were induced by tail vein injection of MDA-MB 231 and MCF-7 cells into 5-week-old BALB/c nude mice (Orient Bio, Seongnam-Si, Korea). For inducing MCF-7 model, the mice were ovariectomized and implanted 17β-estradiol pellet (0.72 mg, 60 days release; Innovative Research of America, Sarasota, FL) subcutaneously into the neck. The mice were randomly grouped into two and treated with 0% (vehicle) and 0.02% of HSE in triple distilled water as intragastric injections of 100 µl. Treatment was given for 30 days and then the mice were euthanized and the lungs were removed. The numbers of metastatic tumors on the lung surface were counted. The lungs were fixed with 4% paraformaldehyde. The sections were stained with haematoxylin and eosin (HE), the area of metastatic nodules containing more than six locations were calculated, and the mean area of nodules was recorded as the percentage area of metastases.

### Statistical analysis

The results of the experiments are expressed as mean ± SEM. Statistical analysis was done with student's *t*-test or *ANOVA* test of the SAS program. These were compared by one-way analysis of variance followed by Duncan's multiple range test.

* *HPLC analysis showed many unknown peaks in the extracts. This indicates that the extract contains a complicated mixture of plant compounds involved in biological activity. Analysis of the separated compounds are needed to confirm identification of individually purified compounds. Studies on the biological activity of purified compounds are also needed in another project.*

## Supporting Information

Movie S1 **Live cell microscopy of MDA-MB 231 cells in DMEM incubated without HSE.**
(AVI)

Movie S2 **Live cell microscopy of MDA-MB 231 cells in DMEM containing 10 µg/ml of HSE.**
(AVI)
